# Predictors of oesophageal candidiasis in persons with or without HIV infection

**DOI:** 10.4314/ahs.v18i2.33

**Published:** 2018-06

**Authors:** Martha F Mushi, Nathaniel Ngeta, Mariam M Mirambo, Stephen E Mshana

**Affiliations:** 1 Microbiology and Immunology Department; Weill Bugando School of Medicine, P.O. Box 1464, Mwanza, Tanzania; 2 Department of Internal Medicine Weill Bugando School of Medicine, P.O. Box 1464 Mwanza, Tanzania

In reply,

With great curiosity we have read the letter to editor by Bongomin F. and colleagues^1^ written as a comment to our recent published article in African Health Sciences^2^, we appreciate their interest to our article and inputs. The interest of the study was to investigate the risk factors for esophageal candidiasis (EC). The risk factors are clearly indicated in the original manuscript regardless of HIV status^1^. In addition, HIV was also analysed as one of the risk factor as previously reported elsewhere 3,4. The increase in age was found to protect the patients from developing EC as previous published^5^. In order not to confuse the reader, the odd ratio and 95%confidence interval for age variable in Table 2 in the original article^1^ was reversed to show the decrease in age as the risk of EC and presented in the abstract and content of the original manuscript^1^.

In addition, in this letter to editor we have included results regarding factors associated with EC in HIV negative individuals. Of 554 HIV negative patients, 20(3.6%) had EC. On multivariate logistic regression analysis, increase in age independently protected HIV negative population from getting EC (OR 0.95, 95%CI; 0.92–0.99; p=0.0035), whilebeing female (OR 11.74, 95%CI; 1.55–88.82; p=0.017), alcohol use(OR 20.45, 95%CI; 4.89–86.56; <=0.001), smoking(OR 5.44, 95%CI; 1.18–25.12; p=0.03), antibiotic use(OR 5.2, 95%CI; 1.67–16.21; p<0.005), and having peptic ulcer diseases (OR 8.24, 95%CI; 2.21–30.72; p<0.002) independently predicted EC among HIV negative patients (table 4).

**Table 4:** Risk factors associated with esophageal candidiasis among HIV negative patients attending e ndoscopy unit (n=554)

Variable	Univariate			Multivariate	
	EC n (%)	OR(95%CI)	P value	OR(95%CI)	P
Age*	3931–50	0.99(0.96–1.02)	0.365	0.95(0.92–0.99)	0.035
**Sex**					
Male (307)	11(3.6)	1			
Female (247)	9(3.6)	1.02(0.414–2.496)	0.97	11.74(1.55–88.82)	0.017
**Alcohol**					
No (475)	6(1.3)	1			
Yes (79)	14(17.7)	16.8(6.25–45.35)	<0.001	20.45(4.89–86.56)	<0.001
**Smoking**					
No (515)	13(2.5)	1			
Yes (39)	7(18)	8.45(3.15–22.64)	<0.001	5.44(1.18–25.12)	0.03
**Corticosteroid** **use**					
No (551)	18(3.3)	1			
Yes (3)	2(66.7)	59.2(5.13–683.5)	0.001	−	−
**Clinical** **presentation**					
No (241)	3(1.2)	1			
Yes (313)	17(5.43)	4.56(1.32–15.73)	0.016	3.90(0.90–16.85)	0.068
**Antibiotics use**					
No (448)	9(2.01)	1			
Yes (106)	11(10.38)	5.64(2.27–14.01)	<0.001	5.2(1.67–16.21)	0.005
**Diabetic**					
Negative (542)	14(2.6)	1			
Positive (12)	6(50.0)	37.7(10.81–131.62)	<0.001	−	−
**PUD**					
No (473)	13(2.8)	1			
Yes (81)	7(8.6)	3.35(1.29–8.66)	0.013	8.24(2.21–30.72)	0.002
**Asthma**					
No (550)	18(3.27)	1			
Yes (4)	2(50.0)	29.56(3.94–221.79)	0.001	−	−

PUD is the peptic ulcer diseases; some known variable like use of corticosteroid, having diabetic and asthma were not include in multivariate analysis because the patients with EC were very few which could lead to a very wide confidence interval.

Furthermore, we have noted that, the original published article did not incorporate the corrections which were presented during proofreading, for this reason we have presented the correct table 3 as seen below:

**Table 3:** Characteristics of HIV negative patients with EC

S/number	Sex	Age	Presentation	Alcohol	Smoking	Antibiotic	Diabetic	Cirrhosis	PUD	Asthma
1	Male	20	Hemoptysis	Yes	Yes	No	No	Yes	No	No
2	Female	24	Ep. pain	No	No	Yes	No	No	Yes	No
3	Male	32	Ep. Pain	Yes	Yes	Yes	No	No	No	No
4	Female	30	Ep. Pain	Yes	No	Yes	No	No	No	No
5	Male	47	Dysphagia	Yes	Yes	Yes	Yes	No	No	No
6	Female	17	Dysphagia	No	No	No	No	No	No	Yes
7	Male	40	Ep. pain	Yes	No	Yes	No	No	Yes	No
8	Female	38	Ep. pain	Yes	No	No	Yes	No	No	No
9	Female	47	Ep. pain	No	No	No	Yes	No	No	No
10	Male	77	Ep. pain	Yes	Yes	Yes	No	No	No	No
11	Female	38	Ep. pain	No	No	Yes	No	No	Yes	No
12	Male	34	UGIB	Yes	Yes	No	No	No	Yes	No
13	Female	60	Ep. pain	Yes	No	Yes	No	No	Yes	No
14	Male	28	Ep. pain	Yes	Yes	No	No	No	No	No
15	Male	40	Dysphagia	Yes	No	No	Yes	No	Yes	No
16	Male	36	Ep. pain	Yes	No	Yes	Yes	No	No	No
17	Male	44	UGIB	Yes	Yes	No	No	Yes	No	No
18	Female	62	Ep. pain	No	No	No	Yes	No	No	No
19	Female	33	Ep. pain	Yes	No	Yes	No	No	Yes	No
20	Male	23	Ep. pain	No	No	Yes	No	No	No	Yes

Note; Ep. Pain is epigastric pain and UGIB is upper gastro intestinal bleeding
